# Development of a novel method for the in-situ dechlorination of immovable iron elements: optimization of Cl^−^ extraction yield through experimental design

**DOI:** 10.1038/s41598-021-90006-y

**Published:** 2021-05-24

**Authors:** Marco Veneranda, Nagore Prieto-Taboada, Jose Antonio Carrero, Ilaria Costantini, Aitor Larrañaga, Kepa Castro, Gorka Arana, Juan Manuel Madariaga

**Affiliations:** 1 Department of Analytical Chemistry, University of the Basque Country (UPV/EHU), P.O. Box 644, 48080 Bilbao, Spain; 2 Department of Condensed Matter Physics, Crystallography and Mineralogy, University of Valladolid, Valladolid, Spain; 3 General Research Services (SGIker), University of the Basque Country, Leioa, Spain; 4 Unesco Chair of Cultural Landscapes and Heritage, University of the Basque Country (UPV/EHU), 01080 Vitoria-Gasteiz, Spain

**Keywords:** Analytical chemistry, Infrared spectroscopy, X-ray diffraction, Design, synthesis and processing

## Abstract

The conservation of iron objects exposed to marine aerosol is threatened by the formation of akaganeite, a highly unstable Cl-bearing corrosion phase. As akaganeite formation is responsible of the exfoliation of the rust layer, chlorides trigger a cyclic alteration phenomenon that often ends with the total consumption of the iron core. To prevent this degradation process, movable iron elements (e.g. archaeometallurgical artefacts) are generally immersed in alkaline dechlorination baths. Aiming to transfer this successful method to the treatment of immovable iron objects, we propose the in-situ application of alkaline solutions through the use of highly absorbent wraps. As first step of this novel research line, the present work defines the best desalination solution to be used and optimizes its extraction yield. After literature review, a screening experimental design was performed to understand the single and synergic effects of common additives used for NaOH baths. Once the most effective variables were selected, an optimization design was carried out to determine the optimal conditions to be set during treatment. According to the experimental work here presented, the use of 0.7 M NaOH solutions applied at high temperatures (above 50 °C) is recommended. Indeed, these conditions enhance chloride extraction and iron leaching inhibition, while promoting corrosion stabilization.

## Introduction

The conservation of Built Heritage located near the coastline is constantly threatened by the presence of chlorides^1–3^. As major component of marine aerosols, Cl^−^ ions trigger physical and chemical weathering of Built Heritage materials^4–6^. Focusing on iron-based components (both structural and ornamental), chlorides infiltration promotes the formation of unstable corrosion phases that accelerate deterioration processes^7–11^. Among them akaganeite (FeO_0.883_(OH)_1.167_Cl_0.167_), which chemical structure is characterized by tunnels (filled by chloride ions) parallel to the c-axis of the tetragonal lattice, tends to form low density rust layers^12^, whose high fragility facilitates cracking and exfoliation phenomena^13,14^. If not treated, akaganeite formation triggers a cyclical mechanism that often ends with the total consumption of the iron core^15^. Beyond the deterioration of iron elements directly exposed to the external environment, chlorides can penetrate porous concrete thus reaching steel reinforcing bars (rebar)^16–18^. Knowing that akaganeite is the Fe-corrosion product having the highest coefficient of expansion (akaganeite 3.48 > lepidocrocite 3.03 > goethite 2.91 > hematite 2.12 > magnetite 2.08^19^), the internal pressure generated by akaganeite formation on rebars causes concrete’s cracking and spalling^20–22^. The consequent exposure of rebars to the aggressive external environment accelerates the propagation of reinforcement corrosion, which ends undermining the structural integrity of the building^23^.

To prevent the Cl-driven alteration of iron components, preservation treatments are therefore required to (1) extract Cl^−^ ions penetrated within the iron-corrosion layers and (2) shelter dechlorinated elements from further chloride contaminations. As detailed elsewhere^24^, multiple guidelines are available for the optimal protection and repair of reinforced concrete affected by chloride infiltration^25,26^. For example, one of the most conventional repair strategies consist in the re-passivation of rebars (e.g., by applying surface corrosion inhibitors^27^ or by using electrochemical chloride extraction methods^28^), followed by the replacement of the contaminated concrete with an alkaline material highly resistant to chloride penetration.

With regards to iron elements directly exposed to marine aerosol, dechlorination treatments need to be carried out before the application of paint coatings or corrosion inhibitors^29–31^. In this regards, even though very effective dechlorination strategies have been recently developed (e.g. the immersion in alkaline subcritical fluids^32,33^) the immersion in alkaline baths is the most employed method to stabilize movable iron items. Indeed, besides being cost effective, easy to implement and highly effective, desalination baths also prevent iron leaching and aesthetical alterations of the corrosion layer^34–36^. However, technical problems related to the in-situ application of alkaline solutions difficult its transfer to the treatment of immovable items, as is the case of the ornamental and structural elements of the Built Heritage.

Learning from similar field of research, the in-situ desalination of mortars and masonries is effectively achieved by the use of extractive solutions imbibed in highly absorbent materials (e.g. cellulose pulp, carboxymethyl cellulose and clays^37–40^). Directly applied on the element to be treated, the imbibed wraps allow a controlled iteration of the solution with the contaminated surface, favouring the in-depth solubilization and extraction of soluble salts (including Cl- ions)^41^. In spite of the proven compatibility between common wrapping materials (such as bentonite and sepiolite clays) and strong alkaline solutions^42–45^, the described method has never been applied to the in-situ dechlorination of iron elements.

Filling this gap, a novel research line was defined to evaluate the applicability of absorbent wraps imbibed with alkaline solutions, for the in-situ dechlorination of immovable iron elements. In this framework, the present manuscript investigates the real effects entailed by the main alkaline solutions used for the dechlorination of iron artefacts and defines the optimal values ensuring the best results in term of Cl^−^ extraction, iron leaching inhibition and corrosion stabilization.

In this regards, it is important to underline that the majority of alkaline dechlorination baths are nowadays carried out by using NaOH-based solutions^35,36,46^. In spite of their well-recognized efficacy, literature proves that several additives have been recently proposed to further improve the stabilization capability of NaOH baths. For example, sodium sulphite (Na_2_SO_3_) has been used as additive to increase the desalination capability of the solutions^47,48^. Similarly, the synergic use of NaOH and ethylenediamine has been tested to improve the iron leaching inhibition of the alkaline bath^49^,which represents one of the main side effects of immersing metallic artefacts in aqueous solutions. Numerous researchers also experimented with the deoxygenation of alkaline baths to limit the development of new corrosion phases during treatment^50^. Indeed, is it well known that during immersion, oxygen can react with the iron surface leading to the onset of new degradation products. In addition to the use of additives, it is well known that the efficacy of NaOH solutions can be strongly enhanced by increasing their temperature above 50 °C^51^.

Considering the large number of variables to take into account, the present work focused on (1) evaluating their single and synergic effect on the efficacy of NaOH-based baths, and (2) optimizing the extraction yield of the dechlorination solution to be used for wraps’ imbibition. To achieve these goals, we made use of experimental designs, which advantages in these kinds of studies has been proven in numerous works^52–54^.

## Materials and methods

### Alkaline baths comparison and optimization

Even though literature shows many scientific articles focused on evaluating the effects entailed by those single variables/additives on the efficacy of NaOH-based baths, only a few studies focused on maximizing the extraction yield of the proposed treatments. In this context it must be also underlined that very little is known about the synergic effects (positive and negative) produced by the simultaneous use of two or more variables/additives. In order to fulfil this gap of knowledge, the real effects entailed by the mentioned variables are here investigated by means of experimental design. As described elsewhere, this is a statistical tool that allows to identify (with the minimum number of experiment) the effect entailed by single variables on predetermined responses, as well as monitor the possible positive/negative interactions between variables^55^.

In detail, a screening experimental design^56^ was carried out to: (1) identify the variables having the highest influence in both, Cl^−^ extraction and Fe leaching inhibition, and (2) monitor the possible positive/negative interactions between variables. Taking into account the above mentioned NaOH-based baths, a full factorial design was performed taking into consideration 5 variables and 2 levels (low and high values, codified as − 1 and + 1 respectively) without centre points (see Table 2). Afterwards, the influence of the most important variables was investigated and optimized by central composite design (CCD) combined with response surface methodology (RSM). As explained by Torrades et al.^57^ this method analyzes each variable at 5 different levels. The low, centre and high levels of each variable are designated as − 1, 0 and + 1, respectively, while − 1.68 and + 1.68 codified levels have the purpose of predicting the response functions outside the cubic domain (see Table 4). Each experiment of the proposed design was carried out in triplicates. In both cases, the Unscambler software (CAMO ASA, Norway) was employed for data interpretation.

For both experimental designs, treatments were carried out by immersing 0.25 g of powdered akaganeite in 50 ml of alkaline solution. The ratio between sample weight and solution volume of 1/200 was established to avoid saturation phenomena. During treatment, the vessels containing the mixture of akaganeite and NaOH solutions were sealed to prevent the evaporation of the liquid phase and the contact with atmospheric oxygen. Temperature was controlled using a Heraeus Function Line heating oven. With regards to solutions deoxygenation, a two steps protocol was applied. In brief, a preliminary deoxygenation was carried out by immersing the solvent container in an ultrasonic bath (Ultrasons-H, JP Selecta, Spain) for 1 h. Afterwards, Helium sparging was additionally performed, by bubbling the noble gas through the solutions for 5 min^58^ .

### Akaganeite synthesis

Even though experimental designs have been successfully used in several field of study^59–61^, reliable statistical results are difficult to attain through the study of heterogeneous samples (i.e. real iron objects and rust samples). Therefore, the experiments presented in this work have been carried out by treating pure samples of akaganeite synthesised in the laboratory. The adopted strategy had the purpose of ensuring repetitive results and simplifies the detection of corrosion phase transformations. Akaganeite synthesis was performed by following the method proposed by Reguer et al.^62^, which consists in heating (at a constant temperature of 70 °C) an aqueous solution of FeCl_3_ 0.1 M (reagent grade, supplied by Sigma Aldrich, purity of 97%) in a sealed baker for 48 h. To remove the majority of free Cl^−^ ions, the precipitated powder was then subjected to a thorough washing process using Milli-Q water. The solid phase was finally collected after centrifugation and dried at room temperature for 7 days.

### Iron corrosion characterization

The molecular composition of the final product was characterized by means of the Analytical Xpert PRO X-Ray Diffractometer (XRD, PANalytical, Netherlands). The XRD system is equipped with a copper tube, a vertical goniometer (Bragg–Brentano geometry), a programmable divergence slit, a secondary graphite monochromator and a Pixcel detector. The measurement conditions were set at 40 kV, 40 mA and a scan ranging between 5 and 70 °2theta. Diffractograms interpretation was performed using WinPLOTR software, by comparison with the PDF-4 standards database^63^.

### Treated samples characterization

After each experiment, the solid phase (treated akaganeite) was separated from the NaOH-based solution by centrifugation. The quantitative analysis of extracted chlorine was performed by using a Dionex ICS 2500 ion chromatograph (Thermo Scientific, USA) equipped with an ED50 conductivity detector and an AS 40 auto sampler. Before analysis, the NaOH-based solutions were subjected to a dilution process using Milli-Q water. Analyses were carried out by using an IonPac AS23 (4 × 250 mm) column, an IonPac AG23 (4 × 50 mm) pre-column, 4.5 mmol/L Na_2_CO_3_/0.8 mmol/L NaHCO_3_ mobile phase (reagent grade, both supplied by Sigma Aldrich, purity of 99,9%), 25 mA suppression current and a flow of 1 mL min^−1^. Extracted Cl^−^ ions were quantified through an external calibration curve, while data processing was performed using the 6.60-SPIA CHROMALEON software (Dionex Corporation, USA).

To assess the possible lixiviation of iron during samples treatment, a NexION 300 Inductively coupled Plasma—Mass Spectrometry system (PerkinElmer, USA) was employed. Before analysis, NaOH-based solutions were diluted (Milli-Q water), while a standard solution of HNO_3_ (tracepur grade, supplied by Merk, Germany) was used to reach the optimal acidity value. The quantification of ^56^Fe isotope was performed under the following experimental conditions: nebulizer flow of 0.9–1.0 mL min^−1^, plasma flow of 18 mL min^−1^ and radio frequency power of 1400 W. Argon with a purity of 99.995% was provided by Praxiar (Spain). Analyses were carried out inside a clean room (class 100) and quantitative data were obtained by means of external calibration curve, using a 1000 mg L^−1^ standard solution (Specpure, Plasma standard solution, Germany). Data acquisition and interpretation was done using the NexION 1.5 software (Perkin Elmer, USA).

To verify the possible transformation of akaganeite into more stable phases, qualitative analyses were carried out with a Fourier transform infrared spectroscopy (FTIR) system working in transmittance mode. Concretely, a 6300 FTIR spectrophotometer (Jasco, Japan) composed of a Ge on KBr beamsplitter, a Michelson interferometer and a DLaTGS detector was used. To prepare the pellets, 0.5 mg of treated akaganeite was mixed with 170 mg of dry KBr (FTIR grade, supplied by Sigma-Aldrich, purity > 99%), milled in an agate mortar and pressed under 10 tons/cm^2^ for 8 min. All analyses were carried out in the middle infrared region (from 4000 to 400 cm^−1^) recording 64 scans at 4 cm^−1^ spectral resolution. The qualitative analysis of the collected spectra was done using the Omnic software version 7.2 (Thermo Nicolet, USA). After completing the FTIR analysis of all solid samples, the PALME software (Program d'AnaLyse vibrationnelle de spectres de MElanges à partir de spectres purs) developed by the LADIR Laboratory (now MONARIS, Pierre et Marie Curie University, France) was employed to semi-quantify the detected iron corrosion phases^64^. The reliability of this tool regarding the study of iron corrosion phases is deeply described in a dedicated work^65^.

## Results and discussion

### Akaganeite synthesis

**Figure 1:**
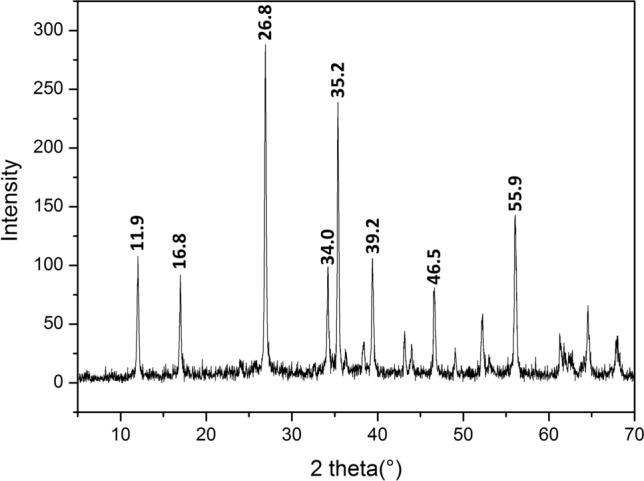
XRD diffractogram collected from synthesized akaganeite.

As represented in Fig. 1, the X-ray diffractogram obtained from the analysis of synthetic akaganeite displays main peaks at 26.8 (relative intensity of 100%), 35.2 (79.2%), 55.9 (49.5%), 11.9 (35.9%), 39.2 (34.8%), 34.0 (31.9%), 16.8 (29.6%) and 46.5°2Theta (26.7%), which fit with the akaganeite reference standard from the PDF-4 database (00–042-1315).

In order to correctly identify and semi-quantify the potential secondary corrosion products formed during treatment, the synthetized material need to be mineralogically pure. To verify this aspect, the whole list of detected diffractometric peaks was analysed in detail. As summarized in Table 1, positions and relative intensity values of all detected peaks perfectly matched the reference 00-042-1315, thus proving the synthesis of pure akaganeite. In this sense, Table 1 also provides the corresponding basal planes of each detected signal.

**Table 1:** List of peaks detected from the XRD analysis of synthetic akaganeite.

Peak position (°2Theta)	Intensity (counts)	FWHM (°2Theta)	d-spacing (Å)	Relative intensity (%)	Corresponding basal planes (h, k, l)
11.9	97.3	0.20	7.45	35.9	1, 0, − 1
16.8	80.2	0.18	5.27	29.6	0, 0, 2
26.8	270.9	0.15	3.33	100.0	1, 0, − 3
34.0	86.4	0.18	2.63	31.9	0, 0, 4
35.2	214.5	0.18	2.55	79.2	2, 1, − 1
38.2	20.7	0.26	2.35	7.6	2, 0, 4
39.2	94.4	0.13	2.30	34.8	3, 1, 0
43.0	33.1	0.20	2.10	12.2	3, 1, − 2
43.8	17.9	0.31	2.07	6.6	5, 0, − 1
46.5	72.2	0.20	1.95	26.7	1, 1, − 4
48.9	19.5	0.20	1.86	7.2	4, 0, 4
52.1	43.4	0.20	1.76	16.0	6, 0, 6
55.9	134.2	0.20	1.64	49.5	5, 1, − 2
61.2	25.1	0.26	1.52	9.3	0, 2, 0
64.4	53.2	0.26	1.45	19.6	5, 1, − 4
67.8	27.0	0.37	1.38	10.0	3, 2, − 1

Besides mineralogical purity, the synthetized akaganeite has to be highly crystalline so as to ensure treatment results similar to those provided by real rust product. Therefore, the X-ray diffractogram was also used to determine the average size of the crystalline domain (mean coherence length (MCL)) of the sample, which value was extracted from the broadening of the main diffractometric signal using the Scherrer equation (Eq. 1)^66^:

1 $$\beta_{hkl} = {{k \cdot \lambda } \mathord{\left/ {\vphantom {{k \cdot \lambda } {L_{hkl} \cdot \cos \theta }}} \right. \kern-\nulldelimiterspace} {L_{hkl} \cdot \cos \theta }}$$

where β_hkl_ is the broadening of the main diffraction line measured at half the line maximum intensity (FWHM) taking in to account instrumental contribution (β_Inst_ = 0.1°), λ is the X-ray wavelength, L_*hkl*_ is the crystal size and θ is the diffraction angle and, K is the Scherrer shape factor (K = 0.9 was used for the calculations).

By taking into consideration the FWHM of the main peak detected at 26.8°2theta, the mean crystallite size was calculated to be around 80 nm. This value was found to be in accordance with the MCL values calculated from the XRD analysis of real akaganeite-rich rust samples collected from iron archaeological artefacts deeply analyzed in previous works (between 20 and 100 nm)^15,65^.

### Screening experimental design

According to the review on alkaline dechlorination baths provided in the introduction section, NaOH (A), Na_2_SO_3_ (B), ethylenediamine (C), temperature (D) and deoxygenation (E) were the variables selected for the screening experimental design. The defined low (-1) and high (+ 1) levels listed in Table 2 were chosen according to bibliographic data^35,36,46–51^.

**Table 2:** Variable and levels used in the full factorial screening design.

	Variables	Codified levels	
		− 1	1
A	NaOH (M)	0	0.5
B	Na_2_SO_3_ (M)	0	0.05
C	Ethylenediamine (% v/v)	0	5
D	Temperature (°C)	25	70
E	Deoxygenation	No	Yes

All the experiments were started at the same time and, after 48 h of treatment, solid and liquid phases were separated by centrifugation. After filtering, washing and dilution processes the amount of extracted Cl^−^ and leached ^56^Fe were quantified by IC and ICP-MS respectively. Quantitative results obtained from each experiment are reported in Table 3.

**Table 3:** Design matrix and results of the 2^5^ full factorial screening design.

Experiments	Codified variables					Responses	
	A	B	C	D	E	Cl (mg/L)	Fe (μg/L)
T01	− 1	− 1	− 1	− 1	1	6037	119.1
T02	1	− 1	− 1	− 1	1	18,090	13.5
T03	− 1	1	− 1	− 1	1	17,956	27.4
T04	1	1	− 1	− 1	1	18,338	13.9
T05	− 1	− 1	1	− 1	1	20,251	2.4
T06	1	− 1	1	− 1	1	19,065	13.6
T07	− 1	1	1	− 1	1	20,752	11.7
T08	1	1	1	− 1	1	19,251	18.5
T09	− 1	− 1	− 1	1	1	7105	151.6
T10	1	− 1	− 1	1	1	31,573	5.9
T11	− 1	1	− 1	1	1	22,418	26.1
T12	1	1	− 1	1	1	32,063	14.7
T13	− 1	− 1	1	1	1	21,237	4.1
T14	1	− 1	1	1	1	33,265	7.5
T15	− 1	1	1	1	1	23,145	21.0
T16	1	1	1	1	1	33,435	7.9
T17	− 1	− 1	− 1	− 1	− 1	6021	254.2
T18	1	− 1	− 1	− 1	− 1	35,081	8.9
T19	− 1	1	− 1	− 1	− 1	17,554	23.8
T20	1	1	− 1	− 1	− 1	18,033	21.4
T21	− 1	− 1	1	− 1	− 1	20,820	12.5
T22	1	− 1	1	− 1	− 1	18,998	24.4
T23	− 1	1	1	− 1	− 1	20,757	18.4
T24	1	1	1	− 1	− 1	18,972	42.7
T25	− 1	− 1	− 1	1	− 1	6826	161.8
T26	1	− 1	− 1	1	− 1	31,981	14.3
T27	− 1	1	− 1	1	− 1	22,665	33.9
T28	1	1	− 1	1	− 1	32,626	9.5
T29	− 1	− 1	1	1	− 1	22,124	14.7
T30	1	− 1	1	1	− 1	34,065	11.8
T31	− 1	1	1	1	− 1	23,310	16.1
T32	1	1	1	1	− 1	32,889	18.6

The results reported in Table 3 were used to run the analysis of effects. In this sense, the table provided as supplementary information (Table SI1) describes how the variables and their two-way interactions influence both Cl^−^ extraction and Fe lixiviation responses (standard cut-off α = 0.05 was set).

In brief, important inferences about Cl^−^ extraction were deduced from *p* value determination. Statistical results of single variables effects proved that NaOH and temperature have significant positive main effect on the yield of Cl^−^ extraction (*p* value of 0.0001 and 0.0005 respectively). Ethylenediamine also had a positive influence on this variable response (*p* value = 0.0135) while Na_2_SO_3_ and deoxygenation shown low (*p* value = 0.0475) and no significant (*p* value = 0.3065) influence, respectively. Regarding the synergic effects of variables, analysis of variance proved that the two-way interaction of NaOH with Na_2_SO_3_ (A-B *p* value = 0.0475) and ethylenediamine (A-C *p* value = 0.0135) had a negative influence on Cl^−^ extraction. On the contrary, the synergic effect shown by NaOH and temperature (A-D *p* value = 0.0036) was expected, as previous laboratory studies demonstrated chloride extraction is enhanced when alkaline media are applied at high temperatures^67^. The analysis of variance provided the β-coefficients of main effects and 2-way interactions of the selected variables. The determined β-values were therefore employed to write the regression equation of Cl^−^ response:

$${\text{R}}\,\left[ {{\text{Cl}}^{ - } } \right] = {22}0{8} + {464}.{\text{8A}} + {13}0.{\text{1B}} + {181}.{\text{2C}} + {358}.{\text{6D}}{-}{24}0.{\text{8AB}} - {237}.{\text{6AC}} + {249}.{\text{6AD}}\;\left( {{\text{mg}}/{\text{L}}} \right)$$

The obtained model presents a square correlation coefficient (R^2^) of 0.976, which indicates that the 97.6% of the extraction variability is explained by the model.

Regarding Fe lixiviation results, the single variables NaOH (*p* value = 0.0022), Na_2_SO_3_ (*p* value = 0.0083) and ethylenediamine (*p* value = 0.0022) negatively affected the concentration of Fe in the solutions, which suggests their active role in the inhibition of lixiviation process. On the contrary, the synergic effect of A-B (*p* value = 0.0037), A-C (*p* value = 0.0012) and C-D (*p* value = 0.0028) variables increased iron lixiviation. Table SM1 shows the null influence of temperature and deoxygenation on the iron leaching. As showed below, the β-coefficients determined by ANOVA were employed to write the regression equation of Fe response:

$${\text{R}}\,\left[ {{\text{Fe}}} \right] = {35}.{18}{-}{2}0.{\text{37A}}{-}{15}.{\text{46B}} - {2}0.{\text{44C}} + {19}.0{\text{1AB}} + {23}.{\text{87AC}} + {2}0.0{\text{9BC}}\;\left( {\upmu {\text{g}}/{\text{L}}} \right)$$

The square correlation coefficient (R^2^ = 0.984) corroborated from a statistical point of view the validity of the obtained model.

### Optimization experimental design

After data interpretation, a second experimental design was carried out to optimize the Cl^−^ extraction feature of the alkaline desalination bath. According to the results obtained from the screening design, NaOH and temperature parameters were selected for their remarkable positive effect on the Cl^−^ variable response. Regarding Na_2_SO_3_ (B) and ethylenediamine (C) variables, the Cl^−^ regression equation displayed above clearly shows that their single variable influence have a weak positive effect on chloride extraction (βB =  + 130.1 and βC =  + 181.2). However, their synergic effect with NaOH (A) (βAB = − 240.8 and βAC = − 237.6) produces a considerable negative effect. From those results, it was deduced that B and C variables has a positive influence only when their concentration in the solution is very low (below 0.0025 M for Na_2_SO_3_ and 2.5% v/v for ethylenediamine). As the use of inappropriate concentrations of the two compounds may trigger remarkable side effects on the extraction capacity of bath solutions, they were excluded from the optimization design. Similarly, deoxygenation was also excluded, since analysis of variance proved that it has no significant effect on either of the two responses under study. In addition to NaOH and temperature, the variable of time was now included in the optimization experimental design. This parameter was not considered in the screening study since several research works already proved its determinant influence in the process of desalination^68^.

As displayed in Table 4, three selected variables were used to develop a central composite design (CCD). Concerning NaOH, as the pH of standard desalination baths is around 13.5 (reached by using 0.5 M NaOH solutions^46^), molarity values that goes from 0.01 to 1 were considered so that to cover the pH range between 12 and 14. Considering the boiling point of NaOH solutions is close to 100 °C^69^, the maximum temperature value was precautionary set to 94 °C. With regards to the parameter of time, it is well known that the treatment of iron surfaces can last up to several months depending on the thickness of the corrosion layers^70^. However, as this work focuses on the study of fine powdered akaganeite, the duration of the experiments could be reduced to the order of a few days. According to preparatory experiments the maximum duration of the treatment was set to 124 h, this being the minimum time necessary to trigger the conversion of akaganeite into other mineral phases (as suggested by the colour change of the iron-powder in the solution).

**Table 4:** Variables and levels used in the central composite optimization design.

	Factors	Codified variables				
		− 1.68	− 1	0	+ 1	+ 1.68
A	NaOH (M)	0.01	0.2	0.5	0.8	1
B	Temperature (ºC)	26	40	60	80	94
C	Time (h)	6	30	65	100	124

As shown in Table 5, 6 centre point experiments were performed to evaluate the repetitiveness of desalination baths. Considering that each operation was carried out in triplicates, the centre composite design matrix was finally composed of 48 experiments. After treatment, the amount of Cl^−^ extracted and Fe lixiviated by each solution was then quantified by IC and ICP-MS systems respectively (see Table 5). Surprisingly, several treated samples showed a chromatic variation. Considering that this phenomenon is linked to the formation of new iron-phases^70^, the PALME-FTIR method was employed for the molecular semi-quantification of all solid samples^65^.

**Table 5:** Design matrix and results of the central composite optimization design.

Experiments	Codified variables			Responses				
	A	B	C	Cl (mg/L)	Fe (μg/L)	Hematite % (w/w)	Goethite % (w/w)	Akaganeite % (w/w)
T01	− 1.68	0	0	40,953	149.7	0	3.0	97.0
T02	− 1.68	0	0	39,892	122.0	0	2.9	96.1
T03	− 1.68	0	0	39,545	140.1	0	2.7	97.3
T04	1.68	0	0	39,544	9.5	0	98.5	1.5
T05	1.68	0	0	63,756	6.2	0	99.4	0.6
T06	1.68	0	0	65,474	5.0	0	98.7	1.3
T07	0	− 1.68	0	38,709	63.0	0	3.3	96.7
T08	0	− 1.68	0	38,966	93.0	0	3.6	96.4
T09	0	− 1.68	0	39,615	59.1	0	2.9	97.1
T10	0	1.68	0	46,537	41.8	3.2	96.8	0
T11	0	1.68	0	51,511	75.4	3.1	96.9	0
T12	0	1.68	0	59,335	75.3	2.5	97.5	0
T13	0	0	− 1.68	49,472	38.0	0	34.3	65.7
T14	0	0	− 1.68	50,070	25.9	0	34.3	65.7
T15	0	0	− 1.68	51,993	33.0	0	34.2	65.8
T16	0	0	1.68	61,783	9.7	0	100	0
T17	0	0	1.68	63,693	5.7	0	100	0
T18	0	0	1.68	62,442	6.3	0	98.9	1.1
T19	− 1	− 1	− 1	40,425	92.0	0	12.2	87.8
T20	− 1	− 1	− 1	40,047	78.2	0	5.2	94.8
T21	− 1	− 1	− 1	39,257	88.9	0	12.9	87.1
T22	1	− 1	− 1	45,667	15.3	0	40.5	59.5
T23	1	− 1	− 1	45,624	12.8	0	36.5	63.5
T24	1	− 1	− 1	46,289	12.7	0	39.3	60.7
T25	− 1	1	− 1	40,832	116.4	0.7	1.3	97.8
T26	− 1	1	− 1	42,249	106.9	0.6	0.5	98.9
T27	− 1	1	− 1	42,240	101.3	0.6	0.6	98.8
T28	1	1	− 1	45,860	60.6	26	34.6	39.4
T29	1	1	− 1	44,403	68.0	25.3	33.8	40.9
T30	1	1	− 1	47,244	57.0	57.3	40.6	2.1
T31	− 1	− 1	1	39,475	130.4	0	3.5	96.5
T32	− 1	− 1	1	39,885	121.7	0	2.6	97.4
T33	− 1	− 1	1	39,231	93.7	0	4.0	96.0
T34	1	− 1	1	53,389	23.1	0	77.7	22.3
T35	1	− 1	1	54,803	21.1	0	77.5	22.5
T36	1	− 1	1	53,075	26.9	0	76.5	23.5
T37	− 1	1	1	41,434	160.3	0.6	0	99.4
T38	− 1	1	1	41,230	88.0	0.9	0.5	98.6
T39	− 1	1	1	41,988	122.5	0.5	0.8	98.7
T40	1	1	1	63,599	79.9	1.2	97.9	0.9
T41	1	1	1	63,716	76.7	1.1	97.3	1.6
T42	1	1	1	65,429	80.5	0.5	99.5	0
T43	0	0	0	67,834	13.0	0	97.4	2.6
T44	0	0	0	63,963	14.5	0	99.4	0.6
T45	0	0	0	64,068	15.2	0	98.3	1.7
T46	0	0	0	65,745	5.0	0	97.7	2.3
T47	0	0	0	67,324	19.3	0	98.7	1.3
T48	0	0	0	63,801	6.5	0	98.0	2.0

As shown in Table 5, the majority of solid samples were partially transformed into goethite. In these cases, alkaline baths were able to remove chlorides from the crystalline structure of akaganeite, triggering its transformation. Mineral interconversion is a well-known feature of the iron oxide/hydroxide system and, as presented in a previous work, the transformation of akaganeite into goethite can be either related to topotactic reaction or a dissolution-reprecipitation mechanism^67^. In addition, FTIR-PALME results verified that dechlorination experiments carried out at high temperatures involved a partial transformation of treated samples into hematite. This phenomenon is consistent with previous studies proving that, more than depending on the pH of the alkaline solution^67^, the transformation of akaganeite into hematite is thermal-induced^23,67^.

Considering that the transformation of akaganeite into more stable phases is of paramount importance for the optimal preservation of iron objects, the CCD was also used to identify the optimal conditions to obtain the highest concentration of hematite and goethite.

Analysis of variance (ANOVA) was first performed to corroborate, from a statistical point of view, the validity of the obtained results. The ANOVA tables for the 5 response surfaces obtained from quadratic models are provided as supplementary information (SI2).

On one side, the R-squared correlation coefficients of Cl^−^ (0.936), Fe (0.924) responses proves that most of the variance was explained by the model. On the other side, the lower correlation coefficients of goethite (0.859), akaganeite (0.878) and hematite (0.569) responses is due to the fact that their relative concentration was estimated using a semi-quantitative method (rather than the quantitative IC- and ICP-based methods used for Cl- and Fe, respectively).

With regards to the repeatability of the experiments, the reproducibility of Cl-extraction data was calculated by averaging the relative standard deviations (measured as percentage) of the three replicates carried out for each CCD operation, obtaining a mean value of 3.29%.

**Figure 2:**
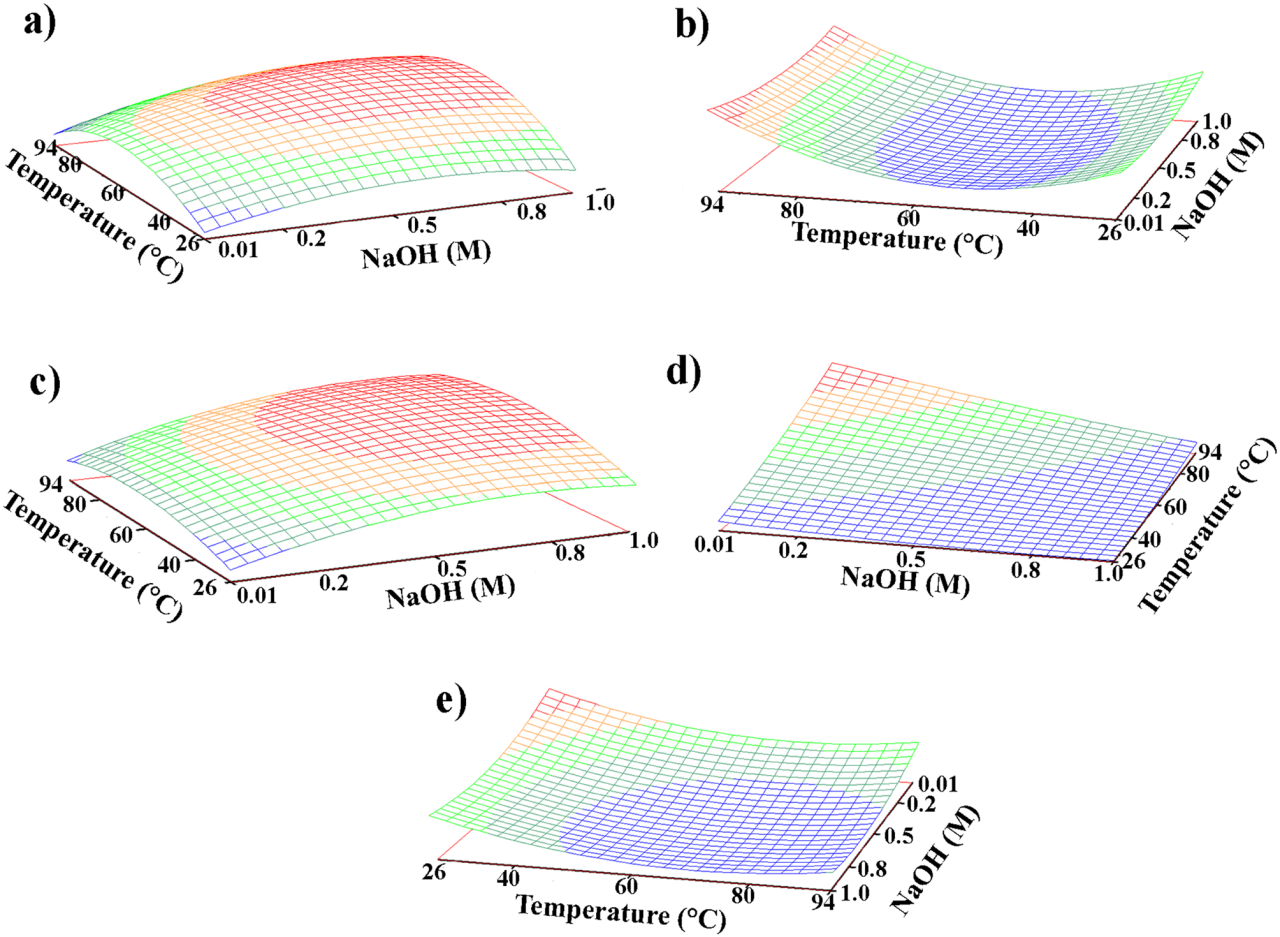
Response surfaces for (**a**) maximum Cl^−^ extraction (t = 85.0 h); (**b**) minimum Fe lixiviation (t = 89.6 h); (**c**) maximum goethite content (t = 99.4 h); (**d**) maximum hematite content (t = 124.0 h); (**e**) minimum akaganeite content (t = 99.4 h).

Afterwards, the response surface methodology (RSM) was employed to identify the input variables settings ensuring the most advantageous responses. Considering the non-linear relationship between input variables and responses, plot results were calculated by using a full quadratic model that considers all interactions and square effects^71^. As shown by the response surface provided in Fig. 2a, the extraction yield of the solution is strongly influenced by both NaOH molarity and temperature. On one hand, Cl-extraction improves significantly when increasing the NaOH molarity from 0.01 M to 0.7 M, while it tends to stabilize at higher concentrations (from 0.7 to 1 M). With regards to the temperature, the highest yields are obtained above 40Cº, reaching the optimal value at 65.7ºC. According to Fig. 2b, the side reaction originating the iron leaching is only slightly affected by the molarity of the solution, while the temperature has a significant impact. In detail, minimum values of Fe-leaching are measured at temperatures between 30 and 70 °C, while maximum values are reached at higher temperatures. Even though the solution’s temperature can be easily controlled during laboratory desalinations, this is often not feasible for in-situ treatments (as is the case of the proposed method, which is based on the application of desalination solution through the mediation of highly adsorbent wraps). However, this result is important as suggests that, to improve the desalination of immovable iron elements, treatments should be carried out during warmer seasons, where temperatures as high as 50 °C are reached in outdoor exposed metal surfaces. The complementarity between the two response surfaces (Fig. 2a,b) is particularly relevant, as it proves that the optimal conditions for chlorides extraction are very similar to those needed to inhibit iron leaching. Similarly, NaOH molarity and temperature settings that enhance Cl^−^ extraction fit to those enabling the transformation of akaganeite into goethite (Fig. 2c). This result was predictable since the optimization of Cl-extraction increases the possibilities of removing Cl^−^ ions within the crystal structure of akaganeite, thus, favouring its transformation into more stable oxyhydroxide phases. The response surface displayed in Fig. 2d proves that minimum NaOH molarity (0.01 M) and maximum temperature (94 °C) are the conditions needed for the transformation of akaganeite into hematite. However, the use of low NaOH molarity/high temperature solutions is strongly discouraged since, as shown in Fig. 2a,b, these conditions cause minimum Cl^−^ extraction and maximum Fe-leaching. Finally, the response surface shown in Fig. 2e (akaganeite content) is in perfect agreement with the results provided in Fig. 2c,d. Indeed, the conditions ensuring the minimum concentration of akaganeite fit with those that guarantee the maximum content of goethite and hematite.

Thanks to response surfaces, the optimal values of temperature, NaOH molarity and time were determined for each considered response (see Table 6).

**Table 6:** Optimal variables conditions obtained by using the surface response.

Variables	Cl max	Fe min	Goethite max	Hematite max	Akaganeite min
Time (h)	85.0	89.6	99.4	124.0	99.4
NaOH (M)	0.71	0.42	0.79	0.01	0.67
Temperature (°C)	65.7	57.2	68.5	94.0	74.1

As can be seen on Table 6, the time necessary to optimize the different responses varies considerably. For example, the maximum transformation of akaganeite into hematite is obtained after 99 h, while the maximum extraction of chlorides is achieved after 85 h. As this result indicates, the optimal duration of desalination treatments does not solely depend on the amount of extracted chlorides, but also on the effect that time has on other important variables, such as iron leaching and corrosion phases transformation. In this context it must be also underlined that, unlike the treatment of powdered synthetic samples (this study), several variables could affect the time needed to reach an effective desalination of real iron items, such as size and shape of the object, porosity of the corrosion system, presence of cracks and fractures, etc. Considering they may strongly influence the efficacy of the treatment under development, these aspects will be deeply evaluated in the next phase of the research line presented in the introduction section.

## Conclusions

This work shows the benefits provided by the use of experimental design to understand the synergic influence of multiple variables in a system. The screening design proved temperature enhances the extraction features of NaOH solutions, while both ethylenediamine and Na_2_SO_3_ showed negative synergic effects. After identifying the most relevant variables, the optimization design was used to improve the solution’s yield. As a result, 0.7 was found to be the optimal molarity for NaOH solutions, as ensures both Cl^−^ extraction enhancement and corrosion stabilization, while inhibiting iron leaching. With regards to the temperature of application, ideal values were measured to be between 60 and 70 °C. Although high temperatures are difficult to be maintained during in-situ treatments, this work indicates in-situ dechlorination treatments should be applied during warmer seasons. As NaOH solutions are compatible with many of the adsorbent materials used for the desalination of Built Heritage’s materials (such as sepiolite and bentonite clays) the in-situ dechlorination of immovable iron objects seems to be feasible. However additional experimental analyses are needed to assess further aspects of the proposed method, as to comprehend the long-term effectiveness of the treatment and evaluate the potential interactions between the rust surface and the adsorbent material.

## Supplementary Information

Supplementary Information.
