# Supplementary material for: Development of a novel method for the in-situ dechlorination of immovable iron elements: optimization of Cl− extraction yield through experimental design
Source: Sci Rep. 2021 May 24;11:10789. doi: 10.1038/s41598-021-90006-y (PMC8144618; doi:10.1038/s41598-021-90006-y)
Supplement: Supplementary file 1 — Supplementary Information. [file 41598_2021_90006_MOESM1_ESM.pdf]

**SUPPLEMENTARY INFORMATION**

# Development of a novel method for the in-situ dechlorination of immovable iron elements: optimization of $Cl^-$ extraction yield through experimental design

Marco Veneranda[1,2], Nagore Prieto-Taboada[1], Jose Antonio Carrero[1], Ilaria Costantini[1], Aitor Larrañaga[3], Kepa Castro[1], Gorka Arana[1], Juan Manuel Madariaga[1,4]

*[1] Department of Analytical Chemistry, University of the Basque Country (UPV/EHU), P.O. Box 644, 48080 Bilbao (Spain). Email: marco.veneranda.87@gmail.com*
*[2] Department of Condensed Matter Physics, Crystallography and Mineralogy, University of Valladolid (Spain).*
*[3] General Research Services (SGIker), University of the Basque Country, Leioa, (Spain).*
*[4] Unesco Chair of Cultural Landscapes and Heritage, University of the Basque Country (UPV/EHU), 01080 Vitoria-Gasteiz (Spain).*

*Table SI1: Codified effects overview. Plus (+) and minus (-) signs describe positives and negatives effects respectively. The significance level of each effect is expressed as a single (weak effect, p-value from 0.05 to 0.01), double (medium effect, p-value from 0.01 to 0.005) or triple (strong effect, p-value ≤ 0.005) sign. Finally, no-significant effects (p-value ≥ 0.05 ) are represented as "ns".*

| **Variables** | **Cl (mg/L)** | **Fe (µg/L)** |
|---|---|---|
| **NaOH (A)** | +++ | -- |
| **$Na_2SO_3$ (B)** | + | -- |
| **Ethylenediamine (C )** | + | -- |
| **Temperature (D)** | +++ | ns |
| **Deoxygenation (E)** | ns | ns |
| **AB** | -- | ++ |
| **AC** | -- | ++ |
| **AD** | ++ | ns |
| **AE** | ns | ns |
| **BC** | ns | ++ |
| **BD** | ns | ns |
| **BE** | ns | ns |
| **CD** | ns | ns |
| **CE** | ns | ns |
| **DE** | ns | ns |

*Table SM2: Analysis of variance (ANOVA) for response surface quadratic models.*

| | Source | Sum of Squares | Degrees of freedom | Mean Square | F-ratio | p-value |
|---|---|---|---|---|---|---|
| Cl- Extraction | **Model** | 4.62E+09 | 9 | 5.14E+08 | 61.508 | <0.0001 |
| | **A-Time** | 1.60E+09 | 1 | 1.60E+09 | 191.222 | <0.0001 |
| | **B-Temperature** | 2.98E+08 | 1 | 2.98E+08 | 35.67 | <0.0001 |
| | **C-NaOH** | 4.67E+08 | 1 | 4.67E+08 | 55.895 | <0.0001 |
| | **AB** | 1.63E+07 | 1 | 1.63E+07 | 1.946 | 0.1711 |
| | **AC** | 2.72E+08 | 1 | 2.72E+08 | 32.511 | <0.0001 |
| | **BC** | 4.27E+07 | 1 | 4.27E+07 | 5.114 | 0.0295 |
| | **AA** | 9.44E+08 | 1 | 9.44E+08 | 112.996 | <0.0001 |
| | **BB** | 1.72E+09 | 1 | 1.72E+09 | 205.557 | <0.0001 |
| | **CC** | 4.54E+08 | 1 | 4.54E+08 | 54.381 | <0.0001 |
| | **Lack of Fit** | 2.41E+08 | 5 | 4.81E+07 | 20.698 | <0.0001 |
| | **Total Error** | 7.67E+07 | 33 | 2.33E+06 | | |
| Fe lixiviation | **Model** | 8.25E+04 | 9 | 9.17E+03 | 20.06 | <0.0001 |
| | **A-Time** | 4.95E+04 | 1 | 4.95E+04 | 108.196 | <0.0001 |
| | **B-Temperature** | 2.39E+03 | 1 | 2.39E+03 | 5.226 | 0.0279 |
| | **C-NaOH** | 1.90E+02 | 1 | 1.90E+02 | 0.416 | 0.523 |
| | **AB** | 2.02E+03 | 1 | 2.02E+03 | 4.424 | 0.0421 |
| | **AC** | 1.09E+02 | 1 | 1.09E+02 | 0.238 | 0.6284 |
| | **BC** | 1.55E+01 | 1 | 1.55E+01 | 0.03396 | 0.8548 |
| | **AA** | 1.82E+04 | 1 | 1.82E+04 | 39.741 | <0.0001 |
| | **BB** | 1.86E+04 | 1 | 1.86E+04 | 40.681 | <0.0001 |
| | **CC** | 1.39E+03 | 1 | 1.39E+03 | 3.038 | 0.0894 |
| | **Lack of Fit** | 1.15E+04 | 5 | 2.30E+03 | 12.945 | <0.0001 |
| | **Total Error** | 5.87E+03 | 33 | 1.78E+02 | | |
| Akaganeite concentration | **Model** | 7.68E+04 | 9 | 8.54E+03 | 30.291 | <0.0001 |
| | **A-Time** | 4.11E+04 | 1 | 4.11E+04 | 145.846 | <0.0001 |
| | **B-Temperature** | 9.46E+03 | 1 | 9.46E+03 | 33.573 | <0.0001 |
| | **C-NaOH** | 6.19E+03 | 1 | 6.19E+03 | 21.977 | <0.0001 |
| | **AB** | 1.66E+03 | 1 | 1.66E+03 | 5.897 | 0.02 |
| | **AC** | 1.96E+03 | 1 | 1.96E+03 | 6.944 | 0.0121 |
| | **BC** | 1.13E+01 | 1 | 1.13E+01 | 0.04026 | 0.842 |
| | **AA** | 1.16E+04 | 1 | 1.16E+04 | 41.167 | <0.0001 |
| | **BB** | 1.14E+04 | 1 | 1.14E+04 | 40.316 | <0.0001 |
| | **CC** | 6.09E+03 | 1 | 6.09E+03 | 21.611 | <0.0001 |
| | **Lack of Fit** | 9.69E+03 | 5 | 1.94E+03 | 62.672 | <0.0001 |
| | **Total Error** | 1.02E+03 | 33 | 3.09E+01 | | |
| Goethite concentration | **Model** | 7.33E+04 | 9 | 8.14E+03 | 25.731 | <0.0001 |
| | **A-Time** | 3.47E+04 | 1 | 3.47E+04 | 109.615 | <0.0001 |
| | **B-Temperature** | 5.91E+03 | 1 | 5.91E+03 | 18.695 | 0.0001 |
| | **C-NaOH** | 9.07E+03 | 1 | 9.07E+03 | 28.671 | <0.0001 |
| | **AB** | 3.56E+02 | 1 | 3.56E+02 | 1.124 | 0.2956 |
| | **AC** | 4.33E+03 | 1 | 4.33E+03 | 13.69 | 0.0007 |
| | **BC** | 3.33E+02 | 1 | 3.33E+02 | 1.053 | 0.3114 |
| | **AA** | 1.28E+04 | 1 | 1.28E+04 | 40.515 | <0.0001 |
| | **BB** | 1.31E+04 | 1 | 1.31E+04 | 41.454 | <0.0001 |
| | **CC** | 6.93E+03 | 1 | 6.93E+03 | 21.9 | <0.0001 |
| | **Lack of Fit** | 1.19E+04 | 5 | 2.39E+03 | 962.237 | <0.0001 |
| | **Total Error** | 8.19E+01 | 33 | 2.48E+00 | | |
| Hematite concentration | **Model** | 2.45E+03 | 9 | 2.73E+02 | 5.576 | 0.0001 |
| | **A-Time** | 2.82E+02 | 1 | 2.82E+02 | 5.771 | 0.0213 |
| | **B-Temperature** | 4.13E+02 | 1 | 4.13E+02 | 8.453 | 0.0061 |
| | **C-NaOH** | 2.73E+02 | 1 | 2.73E+02 | 5.579 | 0.0234 |
| | **AB** | 4.82E+02 | 1 | 4.82E+02 | 9.852 | 0.0033 |
| | **AC** | 4.67E+02 | 1 | 4.67E+02 | 9.561 | 0.0037 |
| | **BC** | 4.66E+02 | 1 | 4.66E+02 | 9.525 | 0.0038 |
| | **AA** | 2.77E+01 | 1 | 2.77E+01 | 0.567 | 0.4562 |
| | **BB** | 6.39E+01 | 1 | 6.39E+01 | 1.308 | 0.2599 |
| | **CC** | 2.77E+01 | 1 | 2.77E+01 | 0.567 | 0.4562 |
| | **Lack of Fit** | 1.19E+03 | 5 | 2.38E+02 | 11.73 | <0.0001 |
| | **Total Error** | 6.69E+02 | 33 | 2.03E+01 | | |
